# A clinical audit of adverse post-nephrectomy outcomes in renal cell carcinoma patients at a tertiary hospital in Queensland, Australia

**DOI:** 10.1007/s40620-024-02173-6

**Published:** 2025-01-07

**Authors:** Julia Chequer de Souza, Venkat Vangaveti, Erik Biros, Andrew J. Mallett

**Affiliations:** 1 Townsville Hospital and Health Service, Douglas, QLD 4814 Australia; 2 College of Medicine and Dentistry, James Cook University, Douglas, QLD 4814 Australia; 3 Institute for Molecular Bioscience, The University of Queensland, Brisbane, QLD 4072 Australia; 4 Townsville University Hospital, Townsville Hospital and Health Service, 100 Angus Smith Drive, Douglas, QLD 4814 Australia

**Keywords:** Renal cell carcinoma, Partial nephrectomy, Radical nephrectomy, Outcomes, Chronic kidney disease

## Abstract

**Background:**

Renal cell carcinoma (RCC) is a common malignancy, and nephrectomy is the mainstay of treatment for non-metastatic disease. The choice of surgery depends on the risks of oncologic recurrence, kidney function decline, and perioperative complications. This study aimed to identify factors associated with adverse post-operative outcomes in RCC patients undergoing nephrectomy at Townsville University Hospital (TUH).

**Methods:**

This was a retrospective, quality assessment study of all adult patients undergoing either open or laparoscopic, partial, or radical nephrectomy for suspected RCC at TUH between January 1, 2016, and December 31, 2020. Patients were identified from the Queensland Health Admitted Data Collection, with a median follow-up time of 39 months post-operatively.

**Results:**

Sixty patients were included; 71.7% underwent radical nephrectomy, and 63.3% were treated with a laparoscopic approach. Adverse kidney function outcomes were identified in 76.7% of patients. In the first 30 days post-nephrectomy, the reduction in estimated glomerular filtration rate (eGFR) in the radical nephrectomy group was more than double that in the partial nephrectomy group (*p* < 0.001). The rise in average serum creatinine post-radical nephrectomy was more than six times that post-partial nephrectomy (*p* = 0.001). This discrepancy in kidney function persisted up to three years post-operatively. No significant differences in RCC recurrence, post-operative cardiovascular events, or mortality were observed between partial nephrectomy and radical nephrectomy (*p* = 0.665, *p* = 1.00, *p* = 0.420).

**Conclusions:**

The balance strongly favours partial nephrectomy despite its underutilisation for patients undergoing nephrectomy for suspected non-metastatic RCC at TUH. Urology teams should weigh the factors favouring radical nephrectomy against the risks of nearly universal renal function decline in this group.

**Graphical abstract:**

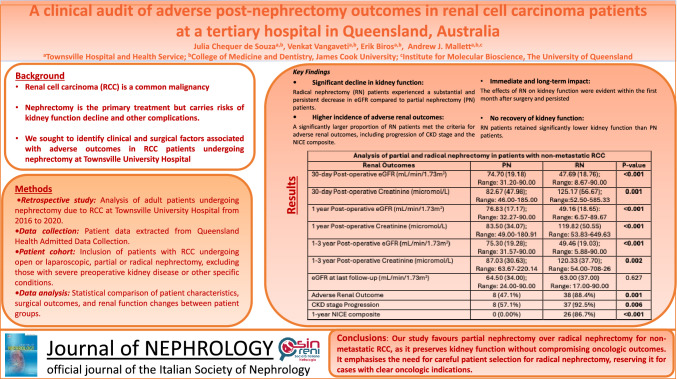

## Introduction

Kidney cancer, particularly renal cell carcinoma (RCC), is the 9th and 14th most common malignancy in men and women, respectively. Surgical management via nephrectomy is the mainstay of treatment for non-metastatic tumours [1]. However, the choice of surgical approach is highly dependent on the relative risk of oncologic recurrence, renal function decline, and perioperative complications [2].

Previous research has demonstrated that Queensland patients who developed chronic kidney disease (CKD) post-nephrectomy had similar rates of adverse events, including progression to end-stage kidney disease (ESKD) and all-cause mortality, to those with primary CKD [3]. Recent data have shown that the burden of ESKD in surgically managed RCC patients in Queensland was 1.9% and 1.0% for radical and partial nephrectomies, respectively [4]. Moreover, rural Queensland patients were found to be less likely to undergo nephron-sparing interventions and, therefore, are at higher risk of adverse kidney function outcomes [5]. Consequently, there is a need for superior risk-stratification resources for urologists managing RCC patients in Queensland to optimise the preservation of kidney function and thus reduce the burden of post-nephrectomy adverse kidney function outcomes.

We aimed to identify patients with RCC who had a nephrectomy at Townsville University Hospital (TUH) over a five-year period, from January 1st, 2016, to December 31st, 2020, and assess the patient, clinical and surgical risk factors for adverse clinical and kidney function outcomes in the short and long term. Understanding the predictors of poorer outcomes in the Townsville Hospital and Health Service (THHS) catchment area is the first step towards improving risk stratification and overall management for its RCC patients.

The primary aim of this study was to conduct a comprehensive analysis of patient factors, clinical characteristics and surgical approaches associated with adverse postoperative clinical and kidney function outcomes in RCC patients at TUH in the short and long term. Secondary objectives included conducting subgroup analysis to identify demographic or surgical factors influencing outcomes and proposing potential novel approaches for risk stratification that integrate local, patient-specific data to facilitate informed clinical and surgical decision-making and enhance the prognostication of patients with RCC undergoing nephrectomy at TUH.

## Materials and methods

### Ethical considerations

The Townsville Hospital and Health Service Audit, Quality and Innovation Review panel reviewed and approved the study as a quality assurance and audit study (THHSAQUIRE1689). A waiver of consent was granted in accordance with the Queensland Hospital and Health Boards Act (Sect. 150; 2011) and Australia’s National Statement on Ethical Conduct in Human Research (NHMRC, 2018) Sect. 2.3.10 as the retrospective quality assurance/audit study analysed previously collected data and constituted negligible risk to participants who had already received standard-of-care.

### Study population, hospital, and health services

This was a retrospective, quality assessment study of all adult patients undergoing open or laparoscopic, partial, or radical nephrectomy for suspected RCC at Townsville University Hospital (TUH) between January 1, 2016, and December 31, 2020. Follow-up data were collected until March 31, 2024. Patients with a preoperative eGFR (CKD-EPI) of ≤ 15L/min/1.73m^2^, venous tumour thrombosis, repeat nephrectomy and solitary or pre-operatively transplanted kidneys were excluded. TUH is the largest healthcare facility (791 inpatient beds) in the Townsville Hospital and Health Service and the only tertiary referral centre in northern Queensland. THHS services an area of 148,000 square kilometres and has a resident population of ~ 230,000, or ~ 4.8% of Queensland’s overall population [6]. The TUH also services patients requiring nephrectomies in the North West Hospital and Health Service, which is comprised predominantly of rural and remote communities. Robotic nephrectomies are not conducted at TUH and, therefore, were not considered in this study.

### Data collection

The THHS Research Data Laboratory identified patients from the Queensland Health Admitted Data Collection (QHADC) using ICD-10 classifications. Chart reviews were conducted for missing data enquiries and data quality checks.

### Variables considered

Data collected for each patient included patient demographics (age, biological sex, Aboriginal and/or Torres Strait Islander status, postcode of primary residence), medical history (smoking, alcohol consumption, hypertension, type 2 diabetes mellitus (T2DM), CKD, chronic obstructive pulmonary disease, cardiovascular disease), histopathological data (tumour stage, grade, histology), kidney function outcomes (average eGFR (CKD-EPI) and serum creatinine pre-operatively and at 30 days, one year, three years and at last follow-up, initiation and duration of kidney replacement therapy [KRT] and kidney transplantation), and clinical outcomes (length of stay, intensive care unit [ICU] admission and length of stay, days to last follow-up, cardiovascular event, RCC recurrence and mortality).

The postcode of primary residence was categorised as metropolitan, inner regional, outer regional, remote, or very remote, using Area of Remoteness Index of Australia (ARIA +) codes, and represented as remote (remote and very remote ARIA + code) and non-remote (metropolitan, inner regional, outer regional ARIA + codes) [6]. eGFR at TUH is calculated using the CKD-EPI equation [7]. As a representation of kidney function, the CKD stage was derived solely from eGFR and used as a convenience estimation of kidney function at all follow-up stages. Cardiovascular disease refers to a record/diagnosis of either coronary artery disease (including myocardial infarction, coronary artery bypass grafting, and percutaneous coronary intervention), stroke (including carotid endarterectomy); peripheral arterial disease (including peripheral arterial disease-related complication such as gangrene, amputation); heart failure or cardiovascular disease-related mortality [8]. Excessive alcohol consumption was defined as a documented history of alcohol consumption above the Australian guidelines to reduce health risks from drinking alcohol. This is defined as no more than ten standard drinks a week and no more than four standard drinks on any day [9]. A parameter representing an adverse renal outcome included the National Institute of Health and Care Excellence (NICE) composite of sustained decrease in eGFR by 15 mL/min/1.73m^2^ or more per year and progression of 1 or more CKD stage [10]. Due to the heterogeneity of follow-up periods between patients, impairing our ability to accurately measure a change in eGFR per year, the NICE composite was measured at one-year post-nephrectomy.

### Statistical analysis

SPSS Statistics 27.0 software package was used for all data analysis (IBM, Armonk, NY), with *p* < 0.05 considered statistically significant. Categorical variables are presented as frequency and percentages. Continuous variables' central tendency and dispersion are presented as the mean and standard deviation for normal (parametric) data or median and interquartile range (IQR) for non-parametric data. Data normality was assessed using the Shapiro–Wilk test. Subgroup analysis was performed for radical nephrectomy patients versus partial nephrectomy patients. Categorical variables were compared using the Chi-square test, Mann–Whitney *U* or Kruskal Wallis test for nonparametric variables, and one-way ANOVA for parametric variables.

## Results

### Demographics and medical history

A total of 60 patients were included in the study. Two-thirds of all patients who underwent nephrectomy for suspected renal cell carcinoma were male. The median age was 61.4 years, with the youngest patient being 35 years and the oldest 88 years old. (Table 1). Ten per cent of patients identified as Aboriginal and/or Torres Strait Islander, and 20.0% resided in remote areas within the THHS catchment area (Table 1). Nearly half of the patients undergoing nephrectomy reported a past or current tobacco smoking history, with 30.0% reporting excessive alcohol consumption at the time of surgery. The commonest pre-operative comorbidity was hypertension (43.3%), followed by T2DM (31.7%), CKD (30.0%), cardiovascular disease (26.7%) and lastly, chronic obstructive pulmonary disease (13.3%) (Table 1).

**Table 1:** Demographics, medical history and clinical characteristics for whole cohort

Data Category	Parameter	Frequency (%)/Median (IQR)
Demographics	Sex	Male: 40 (66.7%) Female: 20 (33.3%)
	Age (years)^	61.35 (12.12); Range: 35–88
	Indigenous Status	Indigenous: 6 (10.0%) Non-Indigenous: 54 (90.0%)
	Remote	12 (20.0%)
Medical History	Type 2 Diabetes Mellitus	19 (31.7%)
	Hypertension	26 (43.3%)
	CKD	18 (30.0%)
	CVD	16 (26.7%)
	COPD	8 (13.3%)
	Smoking	29 (48.3%)
	Excessive alcohol consumption	18 (30.0%)
Clinical Measures	Procedure	
	PN	17 (28.3%)
	RN	43 (71.7%)
	Surgical approach	
	Laparoscopic	41 63.3%)
	Open	19 (36.7%)
	Pre-operative eGFR (mL/min/1.73m^2^)	83.75 (20.54); Range: 14.93–90.00
	Pre-operative creatinine (micromol/L)	78.49 (27.91); Range: 45.00–388.20
	Pre-operative CKD stage	
	1	15 (25.0%)
	2	30 (50.0%)
	3a	3 (5.0%)
	3b	2 (3.3.%)
	4	2 (3.3.%)
	5	0 (0.0%)
	Unknown	8 (13.3%)

Data presented as frequency (%) or median (IQR) except where indicated

^mean (standard deviation)

*IQR* Interquartile Range; *CKD* Chronic Kidney Disease; *PN* Partial Nephrectomy; *RN* Radical Nephrectomy; *eGFR* estimated Glomerular Filtration Rate

### Clinical characteristics

Radical nephrectomies were the most common procedure, accounting for 71.7% of all nephrectomies for suspected RCC at TUH. Roughly two-thirds of nephrectomies were done with a laparoscopic approach (63.3%), with the remaining 36.7% being open nephrectomies (Table 1). The median pre-operative eGFR and serum creatinine were 83.75 mL/min/1.73m^2^ and 78.49micromol/L, respectively. Pre-operatively, half of the patients were in CKD stage II and 11.6% in CKD stage IIIa-IV. The remaining 13.3% did not have accessible or available pre-operative pathology results (Table 1).

### Neoplasm characteristics

Of the 60 patients included in the study, 59 had RCC. Over two-thirds of histopathology results were clear cell RCCs (68.3%), 15.0% were papillary and 10.0% were chromophobe RCCs. The remaining 6.7% of histopathology results included multifocal RCC with leiomyomatous stroma, multilocular cystic RCC, unclassified RCC and benign anastomosing haemangioma (Table 2). The RCC specimens were graded using the Fuhrman or International Society of Urologic Pathologists/World Health Organisation (ISUP/WHO) grading systems. Both showed similar frequencies, with grade 2 RCC being the most common (25.0% and 23.3% respectively), followed by grade 3 (11.7% and 18.3%, respectively). A majority of RCCs were classified as American Joint Committee on Cancer (AJCC) stage 1 (71.1%) (Table 2).

**Table 2:** Neoplasm characteristics, renal and clinical outcomes for whole cohort

Data Category	Parameter	Frequency (%) / Median (IQR)
Neoplasm Characteristics	Histopathology	
	Clear cell	41 (68.3%)
	Papillary	9 (15.0%)
	Chromophobe	6 (10.0%)
	Other	4 (6.7%)
	Fuhrman grade	
	1	3 (5.0%)
	2	15 (25.0%)
	3	7 (11.7%)
	4	1 (1.7%)
	ISUP/WHO grade	
	1	1 (1.7%)
	2	14 (23.3%)
	3	10 (18.3%)
	4	1 (1.7%)
	ACJJ stage	
	1	42 (71.1%)
	2	2 (3.3%)
	3	14 (23.3%)
	4	0 (0.0%)
Renal Outcomes	30-day post-operative eGFR (mL/min/1.73m^2^) ^	54.44 (22.10); Range: 8.67–90.0
	30-day post-operative creatinine (micromol/L)	116.84 (52.50); Range: 46.0–585.33
	30-day post-operative CKD stage	
	1	6 (10.0%)
	2	13 (21.7%)
	3a	15 (25.0%)
	3b	11 (20.0%)
	4	3 (6.7%)
	5	2 (3.3%)
	Unknown	8 (13.3%)
	1-year post-operative eGFR (mL/min/1.73m^2^) ^	56.44 (21.90); Range: 6.57–90.0
	1-year post-operative creatinine (micromol/L)	109.50 (56.0); Range: 49.0–649.63
	1-year post-operative CKD stage	
	1	6 (10.0%)
	2	16 (28.3%)
	3a	18 (30.0%)
	3b	12 (18.3%)
	4	3 (5.0%)
	5	2 (3.3%)
	Unknown	3 (5.0%)
	1–3-year post-operative eGFR (mL/min/1.73m^2^) ^	56.51 (22.17); Range: 5.88–90.0
	3-year post-operative creatinine (micromol/L)	111.00 (52.64); Range: 54.0–708.26
	3-year post-operative CKD stage	
	1	4 (8.3%)
	2	13 (25.0%)
	3a	10 (16.7%)
	3b	11 (18.3%)
	4	1 (1.7%)
	5	2 (3.3%)
	Unknown	16 (26.7%)
	eGFR at last follow-up (mL/min/1.73m^2^)	63.00 (35.00); Range: 17.00–90.00
	CKD stage at last follow-up	
	1	5 (10.0%)
	2	27 (45.0%)
	3a	12 (20.0%)
	3b	6 (8.3%)
	4	8 (15.0%)
	5	1 (0.0%)
	Unknown	1 (1.7%)
	Adverse renal outcome	46 (76.7%)
	CKD stage progression	45 (75.0%)
	1-year NICE composite	26 (43.3%)
	Any KRT	4 (6.7%)
Clinical outcomes	LOS (days)	4.85 (3.0); Range: 2.0–12.0
	ICU admission	11 (18.3%)
	ICU LOS	1.00 (1.00); Range: 1.00–4.00
	Last follow-up (days)	1186.00 (1321); Range: 2.00–2857.00
	RCC recurrence	6 (10.0%)
	Cardiovascular event	9 (15.0%)
	All-cause mortality	8 (13.3%)
	Time to death (days)	870.0 (1536); Range: 428–2459
	RCC-cause mortality	3 (5.0%)

Data presented as frequency (%) or median (IQR) except where indicated

^mean (standard deviation)

*IQR* Interquartile Range; *ISUP/WHO* International Society of Urologic Pathologists/World Health Organisation; *AJCC* American Joint Committee on Cancer; *eGFR* estimated Glomerular Filtration Rate; *CKD* Chronic Kidney Disease; *NICE* The National Institute for Health and Care Excellence; *KRT* Kidney Replacement Therapy; *LOS* Length of Stay; *ICU* Intensive Care Unit; *RCC* Renal Cell Carcinoma

### Post-operative kidney function outcomes

The median follow-up time was 1186 days (~ 39 months), ranging from 2 to 2857 days. In the 30-day post-operative period, the mean average eGFR was 54.44 mL/min/1.73m^2^, and the median average serum creatinine was 116.84micomol/L. During this period, a quarter of patients were in CKD stage II (25.0%), with nearly 70% of patients’ renal function falling between the CKD II-IIIb range and 3.3% of patients falling to CKD stage V (Table 2). The distribution of kidney function in this cohort was similar at one- and three years post-nephrectomy. One year post-operatively, the mean average eGFR was 56.44 mL/min/1.73m^2^, which remained unchanged after three years at 56.51 mL/min/1.73m^2^ (Table 2). Likewise, with average serum creatinine, the median average levels were 109.50micomol/L and 111.00micromol/L at one and three years, respectively. The distribution of CKD staging after one year was like that observed after 30 days, with 30% of patients having CKD stage Ia and an unchanged number of patients with CKD V (3.3%). At three years, the frequency of CKD V remained the same; however, the number of patients with CKD IIIa halved. This may be explained by the fact that over a quarter of patients were lost to follow-up by this time (26.7%), as the frequency of other CKD stages was similar (Table 2). The median eGFR at the last follow-up was 63.0051 mL/min/1.73m^2^, with CKD stage II representing the largest proportion of patients at 45.0%. No patients were identified as being in CKD V at the last follow-up (Table 2).

An adverse kidney function outcome was identified in 76.7% of patients who underwent nephrectomy for suspected RCC at TUH. This included any one of the following: Progression of 1 or more CKD stages (75.0%), the one-year NICE composite (43.3%) or new commencement of acute or chronic KRT (6.7%) (Table 2). One patient received a deceased donor kidney transplant, and another was on the kidney transplant waiting list at the time of data collection.

### Post-operative clinical outcomes

The median length of stay post-nephrectomy was 4.85 days, with the longest being 12 days. The median length of stay did not differ between laparoscopic and open nephrectomy groups (4.00 days vs 5.00 days, *p* = 0.349). Eleven patients were admitted to the ICU, with a median length of stay in the ICU of one day. A recurrence of RCC was confirmed in 10.0% of patients, and 15.0% of patients experienced a cardiovascular event post-operatively. Among all patients, 13.3% died of any cause, nearly a third of which were directly attributed to RCC (Table 2).

### Partial vs radical nephrectomy

On average, radical nephrectomy patients were eight years older than partial nephrectomy patients (63.70 years vs 55.41 years, *p* = 0.016). There were no statistically significant differences in sex, Indigenous status, or remote residence between partial and radical nephrectomy patients (Table 3). Furthermore, there were no differences in the frequency of comorbidities, including smoking and alcohol consumption, between the groups. Overall, radical nephrectomies were strongly associated with poorer kidney function outcomes. Average pre-operative eGFR and serum creatinine were comparable between partial nephrectomy and radical nephrectomy patients (89.40 mL/min/1.73m^2^ vs 80.0 mL/min/1.73m^2^, *p* = 0.100; 75.46micromol/L vs 78.67micromol/L, *p* = 0.583). However, in the first 30 days post-nephrectomy, the reduction in eGFR among the radical nephrectomy group was more than double that of the partial nephrectomy group (47.69 mL/min/1.73m^2^ vs 74.70 mL/min/1.73m^2^, *p* < 0.001). Likewise, the rise in average serum creatinine post-radical nephrectomy was more than six times post-partial nephrectomy (125.17micromol/L vs 82.67micromol/L, *p* = 0.001). The difference in average eGFR and serum creatinine between partial nephrectomy and radical nephrectomy patients remained similar, with significantly lower eGFR and high serum creatinine measured among radical nephrectomy patients in the one- (76.83 mL/min/1.73m^2^ vs 49.16 mL/min/1.73m^2^ *p* < 0.001; 83.5micomol/L vs 119.82micromol.L, *p* < 0.001 respectively) and three-year post-operative periods (79.30 mL/min/1.73m2 vs 49.46 mL/min/1.73m2, *p* < 0.001; 87.03micomol/L vs 120.33micromol/L, *p* = 0.002, respectively). There were no statistically significant differences in eGFR between the two groups at the last follow-up (67.50 mL/min/1.73m^2^ vs 63.00 mL/min/1.73m^2^, *p* = 0.627). A large majority of the radical nephrectomy patients experienced an adverse renal outcome, nearly double that of partial nephrectomy patients (88.4% vs 47.1%, *p* = 0.001). Progression in CKD stage was seen in 92.5% of radical nephrectomy cases, compared to 57.1% in the partial nephrectomy group (*p* = 0.006). No patients in the partial nephrectomy group met the criteria for the NICE composite in the first year post-operatively, whereas 86.7% of patients in the radical nephrectomy group did (*p* < 0.001). Patients in both partial nephrectomy and radical nephrectomy groups underwent KRT; however, there was no statistically significant difference between them (11.8% vs 4.7%, *p* = 0.317) (Table 3).

**Table 3:** Subgroup analysis of partial and radical nephrectomy patients

Data Category	Parameter	PN	RN	p Value
Demographics	Sex	Male: 14 (82.4%) Female: 3 (17.6%)	Male: 26 (60.5%) Female: 17 (39.5%)	0.105
	Age (years)^	55.41 (14.10); Range: 35.0–88.0	63.7 (10.62); Range: 35.0–85.0	**0.016**
	Indigenous status	2 (11.3%)	4 (9.3%)	1.00
	Remote	4 (23.5%)	8 (18.6%)	0.726
Medical History	T2DM	7 (41.2%)	12 (27.9%)	0.319
	Hypertension	6 (35.3%)	20 (46.5%)	0.429
	CKD	3 (17.6%)	15 (34.9%)	0.189
	CVD	2 (11.8%)	14 (32.6%)	0.120
	COPD	1 (5.9%)	7 (16.3%)	0.420
	Smoking	7 (41.2%)	22 (51.2%)	0.485
	Excessive alcohol consumption	6 (35.3%)	12 (27.9%)	0.574
Clinical Measures	Pre-operative eGFR (mL/min/1.73m^2^)	89.40 (13.08); Range: 41.38–90.00	80.00 (26.35); Range: 14.93–90.00	0.100
	Pre-operative creatinine (micromol/L)	75.45 (28.60); Range: 47.83–145.50	78.67 (27.00); Range: 45.0–388.20	0.583
Renal Outcomes	30-day post-operative eGFR (mL/min/1.73m^2^) ^	74.70 (19.18) Range: 31.20–90.00	47.69 (18.76); Range: 8.67–90.00	** < 0.001**
	Difference in mean eGFR (pre-operative vs 30-day post-operative)	14.70 mL/min/1.73m2 decrease	32.31 mL/min/1.73m2, decrease	
	30-day post-operative creatinine (micromol/L)	82.67 (47.98); Range: 46.00–185.00	125.17 (56.67); Range:52.50–585.33	**0.001**
	Difference in median creatinine (pre-operative vs 30-day post-operative)	7.22micomol/L increase	46.50micromol/L increase	
	1-year post-operative eGFR (mL/min/1.73m^2^) ^	76.83 (17.17); Range: 32.27–90.00	49.16 (18.65): Range: 6.57–89.67	** < 0.001**
	1-year post-operative creatinine (micromol/L)	83.50 (34.07); Range: 49.00–180.91	119.82 (50.55) Range: 53.83–649.63	** < 0.001**
	1–3-year post-operative eGFR (mL/min/1.73m^2^) ^	75.30 (19.28); Range: 31.57–90.00	49.46 (19.03); Range: 5.88–90.00	** < 0.001**
	1–3 year post-operative creatinine (micromol/L)	87.03 (30.63); Range: 63.67–220.14	120.33 (37.70); Range: 54.00–708-26	**0.002**
	eGFR at last follow-up (mL/min/1.73m^2^)	64.50 (34.00); Range: 24.00–90.00	63.00 (37.00) Range: 17.00–90.00	0.627
	Adverse renal outcome	8 (47.1%)	38 (88.4%)	**0.001**
	CKD stage progression	8 (57.1%)	37 (92.5%)	**0.006**
	1-year NICE composite	0 (0.00%)	26 (86.7%)	** < 0.001**
	Any KRT	2 (11.8%)	2 (4.7%)	0.317
Clinical outcomes	LOS (days)	6.00 (3.00); Range: 2.00–8.00	4.00 (2.00); Range: 3.00–12.00	0.085
	ICU admission	4 (23.5%)	7 (16.3%)	0.377
	ICU LOS	1.00 (0.00); Range: 1.00–1.00	1.00 (3.00); Range: 1.00–4.00)	0.315
	Last follow-up (days) ^	927.88 (784.29); Range: 7.00–2682.00	1261/74 (790/31); Range: 1.00–2847.00	0.216
	RCC recurrence	1 (5.9%)	5 (11.6%)	0.665
	Cardiovascular event	2 (11.8%)	7 (16.3%)	1.000
	All-cause mortality	1 (5.9%)	7 (16.3%)	0.420
	Time to death (days)	1188 n = 1	779.00 (1846); Range: 428–2459	0.750
	RCC-cause mortality	0 (0.00%)	3 (42.9%)	1.000

Data presented as frequency (%) or median (IQR) except where indicated

^mean (standard deviation)

*IQR* Interquartile Range; *PN* Partial Nephrectomy; *RN* Radical Nephrectomy; *T2DM* Type 2 Diabetes Mellitus; *CKD* Chronic Kidney Disease; *CVD* Cardiovascular Disease; *COPD* Chronic Obstructive Pulmonary Disease; *eGFR* estimated Glomerular Filtration Rate; *NICE* The National Institute for Health and Care Excellence; *KRT* Kidney Replacement Therapy; *LOS* Length of Stay; *ICU* Intensive Care Unit; *RCC* Renal Cell Carcinoma

There was no statistically significant difference between partial nephrectomy and radical nephrectomy patients in the length of stay (6.00 days vs 4.00 days, *p* = 0.085), ICU admission (23.5% vs 26.3%, *p* = 0.377) or ICU length of stay (1.00 day vs 1.00 day, *p* = 0.315). Patients who underwent radical nephrectomy were not any less likely to experience RCC recurrence nor any more likely to experience a cardiovascular event post-operatively (5.9% vs 11.6%, *p* = 0.665; 11.8% vs 16.3%, *p* = 1.000). There was only one recorded mortality in the radical nephrectomy group, which was not related to the patient’s RCC. There were no differences in all-cause or RCC-related mortality between the two groups (5.9% vs 16.3%, *p* = 0.420; 0.00% vs 42.9%, *p* = 1.00, respectively) (Table 3).

A sensitivity subgroup analysis of Indigenous and non-Indigenous, remote, and non-remote patients did not identify any statistically significant differences in clinical characteristics, neoplasm characteristics, or outcomes.

## Discussion

The present study found that compared to partial nephrectomies, radical nephrectomies at TUH for suspected RCC were significantly more likely to lead to adverse kidney outcomes. Almost all radical nephrectomy patients experienced a progression in CKD stage as early as 30 days post-operatively and sustained up to 3 years post-nephrectomy. Despite no mortality, oncologic, or metabolic benefit, over 70% of nephrectomies at TUH are radical, as revealed in the study. These findings must be interpreted within the specific context of Australia, particularly in a tertiary hospital situated in the remote tropical region of North Queensland, which serves a vast and geographically dispersed population. In non-metastatic RCC cases, the decision between radical and partial nephrectomy is influenced by several factors, including tumour size, with radical nephrectomy typically favoured for larger tumours [11]. This likely explains the predominance of radical nephrectomy observed in the local tertiary hospital described in this study. The extensive geography of the region might contribute to delayed access to care, resulting in patients often presenting at more advanced stages of the disease; however, other factors, such as ethnicity, cannot be excluded [12]. Our findings underscore the critical need for early detection programs, such as routine urinalysis—a simple, cost-effective point-of-care test that can be administered in local clinics—to improve kidney function monitoring and enable timely intervention.

Furthermore, the choice between partial and radical nephrectomy often depends on the surgeon's experience and the availability of specialised surgical techniques. Partial nephrectomy represents a more sophisticated surgical procedure than radical nephrectomy and requires a comprehensive multidisciplinary team. In smaller hospitals, the limited number of annual procedures may restrict surgeons' expertise in complex procedures like partial nephrectomy. The Hub and Spoke model, where specialised care is centralised at a large urban hospital (the hub) and supported by smaller regional hospitals (the spokes), can ensure equitable access to high-quality care for patients with renal cell carcinoma. By providing training and support to surgeons at regional hospitals like TUH, the hub can help to improve their skills and expand the availability of specialised procedures [13]. In Australia, the Hub and Spoke model has been implemented in various healthcare regions to enhance access to specialised care [14]. Our study highlights the importance of this model in ensuring that patients in regional areas have access to appropriate surgical options for renal cell carcinoma, regardless of their location.

At TUH, there was no statistically significant difference in RCC recurrence between the partial nephrectomy and radical nephrectomy groups, with the two most common stages for RCCs resected at TUH being AJCC T1 and T3. A Canadian comparative study of nephron-sparing surgery and radical nephrectomy on cancer-specific mortality in patients with T1b RCC concluded that nephron-sparing surgery did provide equivalent cancer control relative to radical nephrectomy and suggested that nephron-sparing surgery should be given equal consideration to radical nephrectomy in patients with T1b lesions [15]. Furthermore, a population-based study of patients with localised T3a RCC in the United States identified that partial nephrectomy and radical nephrectomy groups had comparable cause-specific survival in the fat invasion and the venous invasion cohorts, suggesting that regarding T3a RCC, partial nephrectomy and radical nephrectomy may achieve comparable oncologic outcomes [16]. Overall, the rates of RCC recurrence within the first three years post-nephrectomy at the TUH are 10%. This is relatively low compared to the current understanding that 20–30% of patients with localised disease have recurrence after nephrectomy, although approximately three-quarters of these individuals are identified within the first five years after surgery [17]. This would suggest that at TUH, the risk of kidney function decline should outweigh the risk of RCC recurrence when considering the surgery of choice for localised RCC.

This study found similar median lengths of stay in the hospital for open and laparoscopic nephrectomies. However, laparoscopic nephrectomies have been previously associated with shorter length of stay, less blood loss, lower peri-operative morbidity, and fewer wound complications. This has been observed in both partial and radical nephrectomies for RCC and other indications, including live donation and xanthoglomerulonephritis [18–20].

The difference in post-nephrectomy cardiovascular events between partial nephrectomy and radical nephrectomy patients did not reach statistical significance. It has been previously proposed that compared with partial nephrectomies, radical nephrectomies were more strongly associated with higher rates of cardiovascular events, explained in part by the associated reduction in renal function [21, 22]. For example, a systematic review and meta-analysis involving 4304 RCC-indicated nephrectomy patients demonstrated that partial nephrectomy has an independent protective effect on composite cardiovascular events regarding cardiovascular outcomes [23]. However, results from the European Organization for the Research and Treatment of Cancer (EORTC 30904) randomised trial sparked controversy. This study found that partial nephrectomy did not offer protection against advanced CKD or ESKD and showed a higher rate of cardiovascular mortality. However, the study's conclusions remain debatable due to methodological biases and a substantial loss to follow-up [24]. Other studies have also failed to demonstrate a correlation between radical nephrectomy and increased post-operative adverse cardiovascular events [25, 26]. These results suggest that concerns regarding post-operative cardiovascular events may be better evaluated individually rather than implemented in generalised clinical decision-making strategies.

The mean age for radical nephrectomies at TUH was significantly higher than for partial nephrectomies, consistent with previous multicentre studies, including one in Germany, which found that the median age was higher for radical nephrectomy patients in each age category [27]. Other studies in the United States have also examined the underutilisation of nephron-sparing surgeries for RCC in elderly patients and different population groups, including female and rural patients, with the latter being particularly pertinent to the THHS service area [28, 29]. In addition to older patients having higher susceptibility to cardiovascular disease and CKD, as previously discussed, given the current consensus that older patients are more likely to experience post-operative morbidity and mortality and prolonged length of stay, patients’ age and frailty should be strongly considered when deciding between radical or partial nephrectomy [30].

An important limitation of this study is its retrospective design, incurring the risk of missing data. In addition to ICD-10 codes, keywords were used to ensure incorrectly coded patients were included. Loss-to-follow-up is another factor that has led to missing data on primary outcome variables. Furthermore, it is common practice at the TUH Urology Department for patients to have routine blood investigations conducted at private pathology centres, which may or may not be accessible via the TUH’s electronic medical records system. The risk of missing data was mitigated by data linkage by the SSB data linkage team. The analysis between patient, clinical and surgical factors and post-operative outcomes may have been influenced by confounding factors not accounted for in the study design, such as other co-morbidities, medications, or lifestyle factors. In addition, the impact of the recent pandemic on elective surgery, outpatient follow-up and presentations to the emergency department may also act as a confounding factor. The three-year follow-up period may be insufficient to detect all instances of adverse post-operative outcomes, with previous studies showing some cases of end-stage kidney disease more than four years post-operatively [4]. Although some patients were followed up for five to eight years, failure to attend follow-up appointments and outsourcing pathology results to private pathology laboratories were barriers to data collection beyond the three-year follow-up period. Finally, while our study provides valuable insights into post-nephrectomy outcomes in RCC patients at Townsville University Hospital, the relatively small sample size limits the generalisability of our findings. Larger-scale studies with a more diverse patient population are needed to confirm our results and explore the potential impact of regional factors on outcomes. Additionally, multicentre collaborations can help to increase the statistical power of analyses and identify trends that may not be apparent in single-centre studies.

## Conclusions

The choice to treat RCC with partial or radical nephrectomy is clinically made by weighing the risks of oncologic recurrence, renal function decline, and perioperative complications. At TUH, the risk of adverse renal outcomes is significantly higher among patients undergoing radical nephrectomy, which is consistent with international research. It has been previously demonstrated that radical nephrectomy may pose superior oncologic outcomes with lower recurrence rates; however, this was not observed in the TUH patient cohort. Furthermore, patients at TUH undergoing radical nephrectomy were, on average, older and, therefore, more likely to experience morbidity such as chronic disease and longer length of stay. Overall, in the case of patients undergoing nephrectomy for suspected non-metastatic RCC at TUH, the balance strongly favours partial nephrectomy despite its underutilisation. Therefore, it is recommended that urology teams carefully consider the factors that may make radical nephrectomy more favourable in each case against the risks of near-universal renal function decline in this group.

## Data Availability

The datasets generated and/or analysed during the current study are available from the corresponding author on reasonable request.
